# Hospital History of Cape Town

**Published:** 1924-01

**Authors:** 


### HOSPITAL HISTORY OF CAPE TOWN.

THE past of our own hospitals provides many a quaint story, but we are apt to forget the extraordinary conditions which inevitably prevailed in the earlier colonial days. Dr. Elliott, writing in *South African Medical Record*, tells us the hospital history of Cape Town in its earliest days, when the long voyage from Europe caused great mortality on board, principally from scurvy, and when the colony itself had been established as much for recruiting the health of passengers and crews as for anything else. About 1699, a hospital which could hold over 500 patients arose and is described as being surrounded by a moat. It was single-storeyed, convalescents were always put in a loft if space were required, and only those very ill had bedsteads. All who received permission to go out had to return by 9.30 p.m. The truant was always welcomed in the morning with a good thrashing, "beaten with thin Spanish canes or tied to a pole and thrashed with thick, tarred ropes," according to the extent of his leave-breaking. It must have been discouraging, too, to have your clothes made in Cape Town then, for we read that the person corresponding to the dresser in the venereal wards was also the town tailor. A thousand amazing conditions might be quoted, and it is surprising how long such things lasted, for it was not until 1818 that Dr. Bailey established the Old Somerset Hospital in Prestwich Street. In 1859 the New Somerset Hospital arose, and its predecessor became simply a chronic infirmary. Until 1898 the new institution was a Government hospital run by the Colonial Office. Then it was taken over by a Hospital Board and, with its 370 beds, is now managed by a General Board which supervises all the Peninsular hospitals. At present a new and large general hospital is nearing completion at Groote Schuur.

